# Editorial: Natural extracts as food ingredients: from chemistry to health

**DOI:** 10.3389/fnut.2023.1306307

**Published:** 2023-10-31

**Authors:** Erika Salas, Joana Oliveira, Rosa Perez-Gregorio

**Affiliations:** ^1^Department of Analytical Chemistry, Nutrition and Food Science, Universidad de Salamanca, Salamanca, Spain; ^2^Facultad de Ciencias Químicas, Universidad Autónoma de Chihuahua, Chihuahua, Mexico; ^3^Laboratório Associado para a Química Verde da Rede de Química e Tecnologia (LAQV-REQUIMTE), Department of Chemistry and Biochemistry, Faculty of Sciences, University of Porto, Porto, Portugal; ^4^Department of Analytical and Food Chemistry, Food and Agroecology Institute, University of Vigo, Ourense, Spain; ^5^Department of Analytical and Food Chemistry, Food and Health Omics Group, Galicia Sur Health Research Institute (IISGS), Servizo Galego de Saúde-Universidade de Vigo (SERGAS-UVIGO), Ourense, Spain

**Keywords:** natural extracts, bioactive compounds, nutraceuticals, functional foods, pharmacokinetics

Consumers nowadays are even more concerned about the need to change the Food Systems to be future proof. There is a growing trend to demand novel sustainable, healthy and tasty food aligned with safety and economical regards. The Global Alliance for the Future of Food has a set of seven principles: renewability, resilience, health, equity, diversity, inclusion, and interconnectedness (1). The set of these seven principles in the Food Industry is a groundbreaking challenge. The global increase in the incidence of non-communicable diseases has forced the need for reducing the sugar, salt, and fat food levels. Additionally, there is a general trend to minimize the use of additives while reducing the list of ingredients, which must be easily recognized by consumers as natural. An increasing awareness of the relevance of good nutrition for optimal health and wellness plays a pivotal role in consumer choices.

In this context, the use of natural ingredients is imposing on the food industry as promising tools in the design of novel foods. A growing body of research is being made to characterize novel natural extracts with powerful antioxidant and antimicrobial activities which can be used as alternatives to additives. Furthermore, the health benefits attributed to the consumption of these novel natural ingredients as well as the mechanisms behind are being studied.

However, the chemical composition of natural extracts is complex, depending on intrinsic and extrinsic factors such as the natural source, the geographical origin, the agronomic and processing practices, the culinary processing at home etc. Likewise, there is a lack of standardized methodology for extraction and analysis, which results in a wide range of heterogeneous data. Under this context, establishing a structure-health benefit relationship is still a challenge. Besides, bioactive compounds can molecularly bind to food macromolecules, digestive enzymes, and cell receptors affecting the pharmacokinetics, bioaccessibility, bioavailability, metabolism and ultimately affecting their bioactivities and impact on human health.

This Research Topic encompasses five papers reporting significant findings in research related to the impact of natural extracts in food, contributing to the expansion of knowledge in this field. The included papers cover a diverse spectrum of research, extending from molecules to food. They showcase *in vivo* findings that elucidate the biochemistry and *in vitro* mechanisms supporting their application in the food industry.

The study performed by Yang et al. has highlighted the properties of the cranberry powder to be used as a natural substitute for nitrite addition during the production of fermented sausage. The use of fermented food is increasing given the specific organoleptic properties that arise after the fermentation process. However, the safety of these novel foods must be confirmed. The extensive use of nitrites in the design of meat derived products is a reality that must change. The nitrosamines obtained during nitrites metabolization after food containing nitrites intake have been considered as potentially toxic since they are being related with the incidence of several types of cancer. Under this context, Yang et al. have studied the use of cranberry extracts as natural substitutes to nitrites due to antimicrobial and antioxidant properties combined with the colorful potential. Better quality characteristics (color, texture, and flavor, etc.) were observed in fermented sausage containing the cranberry extract while the antimicrobial activities of this extract lead to effectively inhibit the growth of spoilage microorganisms. Indeed, the great potential to use the cranberry extract in the design of novel fermented sausages was revealed under the framework of this study.

Despite the great potential observed in the use of natural products, there is still a need to understand the bioaccessibility, pharmacokinetics and bioavailability of the main compounds contained within the natural extracts. The review carried out by Wang B. et al. is also included in this Research Topic. This review focused on the spray drying of natural ingredients for DPI (dry powder inhalation) as a drug delivery system, and discussed their synthesis and application. The review highlights the main properties to find the appropriate active ingredients, dosing route, and production process for the reasonable application of natural foods with high-added value and therapeutic value. However, the therapeutic value and activities can be affected by several factors such as the solubility, stability under biological conditions or bioavailability. Therefore, this review aims at summarizing the most suitable preparation methods and application means. In this paper, the principle and synthesis of spray drying technology in the preparation of dry powder inhalers of natural products are reviewed, and some successful applications are analyzed as a first approach to use the natural products in the treatment of respiratory diseases, whose prevalence is growing.

The mechanisms of action of some natural extracts was deciphered through three scientific papers also included in this Research Topic. Lin et al. have tested the traditional Chinese herbal extract, the *Scoparia dulcis* L. extract (SDE) to treat the diabetic retinopathy (DR), a main cause of vision loss in diabetic patients. The impact of the herbal extract on cell viability, apoptosis, and ROS production was studied. Moreover, some biomarkers of oxidative stress were analyzed suggesting the potential use of SDE as a novel nutraceutical in the control of DR. In parallel, Wang H. et al. have studied the prophylactic mechanisms of oat antimicrobial peptides on enteritis by using an *in vivo* mice model. The ability of the oat peptides to modulate the intestinal microbiota of the host was herein unraveled. In this study, rats model of DSS-induced ulcerative colitis was used to evaluate the function of oat antimicrobial peptides (AMPs) in protecting the intestinal mucosal barrier. The oxidative stress has been considered as the main cause of the loss of intestinal integrity leading to a prior cause of inflammation concluding in alterations in the immune system. These results indicate that AMPs can change the intestinal flora in DSS-induced colitis, while the AMPs can reduce the decrease in the content of probiotics caused by DSS destruction, so as to effectively alleviate the destruction of colonic mechanical barrier function. An *in vivo* mice model was also used by the article included in the Research Topic by Eo et al.. This study aims to investigate whether longan fruit extract (LE) supplementation can improve diabetic hyperglycemia through modulation of feeding center located in hypothalamus of db/db T2DM mice. The antidiabetic effect of LE was verified after the intake in mice. LE has promoted proopiomelanocortin neuronal activity while inhibiting agouti-related peptide neurons in the arcuate nucleus of hypothalamus. Longan fruit was highlighted within this study as a potential nutraceutical to treat type-2 diabetes mellitus as well as patients with eating disorders.

Overall, this Research Topic through these five papers covers a great range of multidisciplinary research focused on the great potential to use natural extracts in the Food Industry as a true alternative in the design of resilient, sustainable and healthy food.

## Author contributions

ES: Writing—original draft. JO: Writing—original draft. RP-G: Supervision, Writing—original draft, Writing—review & editing.
